# Using the Behaviour Change Wheel and modified Delphi method to identify behavioural change techniques for improving adherence to smoking cessation medications

**DOI:** 10.1186/s12889-023-16278-3

**Published:** 2023-07-17

**Authors:** Amanual Getnet Mersha, Michelle Kennedy, Parivash Eftekhari, KS Kylie Lee, Penney Upton, Catherine Segan, Melissa A. Jackson, Kirsty Jennings, Gillian Sandra Gould

**Affiliations:** 1 School of Medicine and Public Health, The University of Newcastle, University Drive, Callaghan, Newcastle, NSW 2308 Australia; 2 Hunter Medical Research Institute, Lot 1, Kookaburra Circuit, New Lambton Heights, Newcastle, NSW 2305 Australia; 3 NHMRC Centre of Research Excellence in Indigenous Health and Alcohol, Discipline of Addiction Medicine, Faculty of Medicine and Health, The University of Sydney, Camperdown, Australia; 4 The Edith Collins Centre (Translational Research in Alcohol Drugs and Toxicology), Sydney Local Health District, Sydney, Australia; 5 National Drug Research Institute, Faculty of Health Sciences, Curtin University, Perth, Australia; 6 Burnet Institute, Melbourne, Australia; 7 Centre for Alcohol Policy Research, La Trobe University, Bundoora, VIC Australia; 8 University of Canberra, Health Research Institute, 11 Kirianri Street, Bruce, Canberra, ACT 2601 Australia; 9 Cancer Council Victoria, Victoria, Australia; 10 Centre for Health Policy, Melbourne School of Population and Global Health, University of Melbourne, Victoria, Australia; 11 Hunter New England Local Health District Drug & Alcohol Clinical Services, 670 Hunter Street, Newcastle, NSW 2300 Australia; 12 Drug & Alcohol Clinical Research Improvement Network, 1 Reserve Road, St Leonards, NSW 2065 Australia; 13 Faculty of Health, Southern Cross University, Hogbin Drive, Coffs Harbour, 2450 Australia

**Keywords:** Adherence, Behaviour change wheel, Pharmacotherapy, Nicotine replacement therapy, Smoking cessation

## Abstract

**Background:**

Medication adherence is a crucial component of the pharmacological treatment of smoking. Previous interventions targeted to improve adherence to smoking cessation medications (SCMs) were designed using pragmatic approaches. This study aims to develop a comprehensive intervention strategy to improve adherence to SCMs using the Behaviour Change Wheel (BCW) and a modified Delphi method.

**Methods:**

Recommendations for the design of intervention strategies were based on the BCW guide and six studies conducted by the research team. Factors related to healthcare providers and consumers (person making a quit attempt) that showed associations with adherence were mapped into the Capability, Opportunity, Motivation, Behaviour (COM-B) model, and corresponding intervention functions and policy categories. Interventions were then represented using the Behaviour Change Technique Taxonomy. Finally, a modified Delphi study using 17 experts was conducted to evaluate the nominated strategies using the Acceptability, Practicability, Effectiveness, Affordability, Side-effects, and Equity (APEASE) criteria.

**Results:**

Following a stepped approach, an adherence support wheel was designed to guide implementation strategies and programmes. Thirteen intervention strategies were selected. The selected interventions include providing detailed instructions on how to use SCMs; establishing realistic expectations from SCMs; and providing training for healthcare providers regarding comprehensive smoking cessation care with specifics on the provision of adherence support.

**Conclusion:**

The BCW guide and a modified Delphi were applied successfully to design interventions tailored to improve adherence to SCMs. Improving adherence to SCMs requires a comprehensive intervention approach involving various stakeholders. Future research is needed to assess the effectiveness of the nominated intervention strategies.

**Supplementary Information:**

The online version contains supplementary material available at 10.1186/s12889-023-16278-3.

## Background

Ending the tobacco epidemic is a global issue [1]. In 2019, the global number of smokers reached 1.1 billion, resulting in 7.7 million deaths annually [2]. According to a report by the World Health Organization (WHO), tobacco smoking imposes a financial burden of over US$ 1 trillion each year on healthcare expenses and lost productivity worldwide [3]. The health and economic burden of tobacco smoking demonstrates the urgent need for effective interventions [4]. Smoking cessation medications including nicotine replacement therapy, bupropion, and varenicline are effective cessation aides for people who are assessed as nicotine dependent [5]. Smoking cessation rates are further improved when these medications are provided together with multi-session behavioural counselling (e.g. Quitline, hospital cessation clinic) [5]. These three medications are currently licensed widely throughout the globe for smoking cessation with nicotine replacement therapy the most frequently utilised SCM [6].

While meta-analysis suggests that SCMs are effective, there is variability in the findings of individual trials of SCMs [7, 8]. One of the main predictors of effectiveness is medication adherence [8]. Adherence to SCMs is reported to be inconsistent and low [9–11]. The rate of adherence to nicotine replacement therapy for instance was found to be 61% and 26% among participants of randomised controlled trials and participants of population-based studies [8]. Adherence to SCMs was found to be influenced by a multitude of factors related to the consumer (person making a quit attempt) e.g., perception about the medications; health providers (e.g., lack of skill and knowledge); health facilities (e.g., lack of resources); medications (e.g., side effects, cost) and socio-economic factors (e.g., social support) [12].

Previous clinical trials aimed at improving adherence to SCMs were designed using pragmatic rather than recommended theory-based systematic approaches. In 2019, a Cochrane review was conducted to evaluate the effectiveness of interventions targeted at improving adherence to SCMs [13]. Included studies evaluated motivational interviewing [14]; medication adherence counselling [15]; medication monitoring [16]; provision of feedback [17]; linking medication dosing with other daily routines [18]; identification of individual cues [14]; automated medication adherence calls [15]; and financial incentives [19]. The meta-analysis demonstrated only a slight improvement in adherence and smoking cessation rates. For instance, the mean proportion of prescribed medication consumed over 28 days was 3.9% higher among individuals in the intervention group (provision of information and problem-solving to increase adherence) compared to those in the control group [13].

A systematic approach is needed to develop a more effective intervention strategy to improve clinical outcomes [20]. Using an appropriate theoretical framework to design an intervention is recommended by the UK Medical Research Council’s complex intervention framework [21]. Compared to pragmatic intervention development based on researchers' understanding, a theory-based approach is less likely to be influenced by personal bias and more likely to improve clinical outcomes [22–24]. Designing an effective intervention strategy that may improve adherence to SCMs requires a systematic, theory-based approach. Although previously assessed interventions [14–19] reported a slight improvement in adherence to SCMs and quitting, interventions are not yet systematically described and developed using recommended theoretical frameworks.

In this study, we employed a rigorous approach using the Behaviour Change Wheel (BCW) to identify effective interventions to improve medication adherence and achieve successful smoking cessation [25]. BCW is a comprehensive theoretical framework developed by implementation scientists using 19 frameworks of behaviour change identified in a systematic literature review. BCW has been shown to provide a thorough framework for incorporating several sources of data to inform the selection of intervention strategies [25]. The BCW has three layers (centre, middle and outer). The capability, opportunity, motivation, and behaviour (COM-B) model is at the centre of the wheel [25]. The COM-B model recognises the interaction between three components: capability, opportunity, and motivation in modifying any behaviour. These three components are divided into six subcomponents: psychological capability, physical capability, social opportunity, physical opportunity, automatic motivation, and reflective motivation [25].

In the BCW, components of the COM-B are encircled by a middle layer of nine appropriate intervention functions (education, persuasion, incentivisation, coercion, training, restriction, environmental restructuring, modelling, enablement) and seven policy categories which forms the outer layer (communication, guidelines, fiscal, regulation, legislation, environmental/social planning, and service provision). A further description of behaviour change techniques (BCTs) was developed to define the ‘active ingredients’ of interventions comprising 93 unique BCTs within 16 groups [26].

The BCW model has been successfully utilised in various fields to develop complex and multilevel interventions and has shown promising results in modifying health behaviours. For instance, Gould et al. developed an intervention to train health providers at Aboriginal Medical Services in Australia to improve the provision of smoking cessation care to pregnant women [27]; Cassidy et al. designed an intervention to enhance sexual health service use among students [28]; and, Murphy et al. used the BCW to design interventions to improve the involvement of pharmacists in mental health care [29].

This study uses a theory-based systematic approach to integrating findings from systematic reviews and empirical studies to present a toolbox of intervention strategies to improve adherence to SCMs. The proposed interventions were further refined through a modified consensus-based Delphi study among expert panels. This combined approach provides a rigorous and systematic method for intervention development. It enables a thorough evaluation of interventions, integration of expert opinions, iterative refinement of the intervention design, and maximisation of feasibility, acceptability, effectiveness, and safety. Using this combination of strategies creates evidence-based, practical interventions that align with the specific needs and values of the target population [30, 31].

## Methods

This study used a mixed methods approach to combine evidence from systematic reviews and original studies and culminated in a Delphi study among smoking cessation experts. The study is approved by the University of Newcastle Human Research Ethics Committee, approval number H-2020–0334, and informed consent was taken from the participants.

**Fig. 1:**
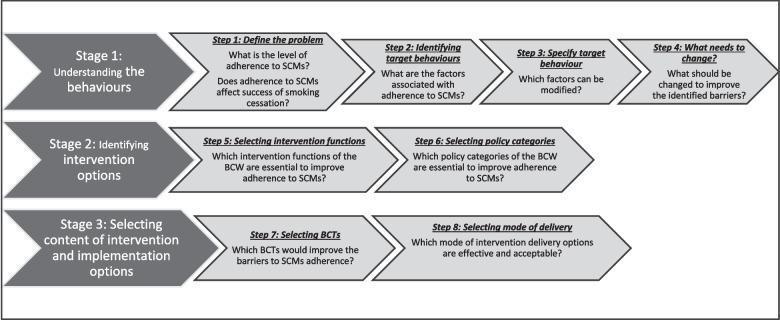
Stages and steps involved in the development of an intervention using the BCW. Adapted from the Behaviour Change Wheel—a guide to designing interventions [37]. (BCW- Behaviour Change Wheel; BCTs- Behaviour Change Techniques; SCMs – Smoking Cessation Medications)

In this study, the BCW was chosen over the theoretical domains framework (TDF) for intervention development because it offers a more comprehensive framework specifically designed for intervention design, whereas the TDF primarily focuses on understanding the determinants of behaviour change. To achieve the objective of the study, we followed the three stages and eight steps recommended in the BCW guide to designing interventions (hereafter called the BCW guide) [25] (Fig. 1). In the first and second stages, we conducted three systematic reviews and three primary studies to understand the rate, impact, and determinants of adherence to SCMs. Details of the studies contributing to stages 1 and 2 have been published [8, 32–36]. Stages 1 and 2 are summarised below.

The final phase (Stage 3) presented here includes the process of selecting the content of the intervention and implementation options using the BCW guide and a modified Delphi study to consult with relevant stakeholders and gain consensus.

### Stage 1: understanding the behaviour

#### (Step 1) Define the problem

We started by evaluating the level of adherence to SCMs and its effect on smoking cessation success using a systematic review [8] and a national survey in Australia [33]. One in four participants was found to be adherent to SCMs [8, 33]. The studies also indicated that adherence improved the success rate of smoking cessation by two-fold (OR = 2.17, 95% CI, 1.34–3.51) [8, 35]. The problem therefore that we seek to address is the low level of adherence to SCMs among individuals making a quit attempt not using SCMs in line with recommendations by healthcare providers.

#### (Step 2) Identifying target behaviours

The target behaviour therefore is to encourage individuals who are making an effort to quit smoking to utilise the SCMs in a manner that aligns with the recommendations provided by healthcare providers. Adherent use of SCMs refers to consistently following the prescribed regimen and instructions for these medications, as advised by healthcare professionals.

#### (Step 3 & 4) Specify target behaviour and identify what to change

Exploration of the barriers and facilitators to adherence to SCMs was conducted using a COM-B informed narrative review [32], and a national cross-sectional study among people who smoke daily and those who had successfully quit in Australia [33]. The factors affecting providing medication adherence support by healthcare providers in Australia were also evaluated [34]. A multitude of factors related to the person making a quit attempt such as forgetfulness, level of nicotine dependence, withdrawal symptoms, perception of the medications, quitting, stress, depression, and social support were found to be associated with adherence to SCMs [32]. The perceived barriers to providing adherence services by healthcare providers were found to be role belief, lack of skill, lack of knowledge, lack of time, and lack of resources [34]. Thus, a range of factors related to consumers, healthcare providers, and healthcare settings were identified as needing to change in order to improve the level of adherence to SCMs (Table 1).

**Table 1:** Factors associated with adherence to SCMs, intervention and policy categories, and Behavioural Change Techniques

Factor category	Factors	COM-B category	Behaviour Change Wheel		Behavioural Change Technique Taxonomy (version 1)	Interventions
			**Intervention functions**	**Policy category**		
Consumer-related factors	✓Self-efficacy ✓Forgetfulness ✓Previous experience of using SCMs	Psychological Capability	Training Education	Communication	BCT 4.1. Instruction on how to perform a behaviour BCT 8.3. Habit formation BCT 15.1. Verbal persuasion about capability	Medication instructions Reminders Self-efficacy Motivating
	✓Extent of nicotine dependence ✓Relapse ✓Medication side effects ✓Withdrawal symptoms	Physical capability	Enablement Education	Communication	BCT 1.4. Action planning BCT 12.4. Distraction	Action plan Distractions
	✓Perception about the effectiveness; safety and need of SCMs ✓Expectation from SCMs	Reflective motivation	Education	Communication	BCT 2.2. Feedback on behaviour 11.1. Pharmacological support	Monitoring and feedback Medication expectation Medication beliefs
	✓Alcohol use ✓Experiencing psychological symptoms such as anxiety ✓Smoking triggers ✓Extent of motivation to quit	Automatic motivation	Restriction Environmental Restructuring Modelling	Communication Regulation Legislation	BCT 1.2. Problem-solving BCT 9.1. Credible source	Alleviating triggers Role Model
	✓Cost of SCMs	Physical opportunity	Incentivisation	Fiscal measures	BCT 10.1. Material incentive (behaviour) BCT 10.2. Material reward (behaviour)	Access Reward
	✓Level of social support ✓Living with a child or children ✓Home-smoking rules	Social opportunity	Environmental Restructuring	Regulation Legislation Environmental/social planning	BCT 3.2. Social support (practical) BCT 5.3. Information about social and environmental consequences	Family as a reminder Impact of smoking on Self and family Health
Health care provider-related factors	✓Lack of skill ✓Lack of knowledge	Physical capability Psychological capability	Training Education	Communication/marketing Guidelines	BCT 6.1. Demonstration of the behaviour BCT 4.1. Instruction on how to perform a behaviour	Training Guidelines
	✓Professional role-belief	Reflective Motivation	Persuasion Incentivisation	Communication/marketing Legislation	BCT 15.1. Verbal persuasion about capability BCT 10.1. Material incentive (behaviour)	Persuasion Incentivisation
	✓Lack of time ✓Lack of resources	Physical opportunity	Environmental Restructuring	Environmental/social planning	BCT 12.5. Adding objects to the environment	Quick reference materials

*SCMs* Smoking cessation medications

### Stage 2: identifying intervention options

#### (Step 5 & 6) Selecting intervention functions and policy categories

The identified factors were mapped to the COM-B. For example, forgetfulness, one of the identified barriers to adherence to SCMs, was mapped to psychological capability as it is associated with the capability of an individual to remember how and when to take the medications. The project team convened multiple meetings to review and identify potential intervention functions and policy categories using the BCW model. The project team is comprised of tobacco treatment specialists, general practitioners, pharmacists, and tobacco researchers. The identified COM-B components were mapped with the relevant intervention functions and policy categories that were most likely to be effective for the required behaviour change. For instance, ‘forgetfulness’, can be improved through education on how individuals can remember to take medications according to provided instructions and it is therefore represented with ‘education’ and ‘communication’ in the intervention function and policy category, respectively (Table 1) (Supplementary table 1).

### Stage 3: Selecting content of intervention and implementation options

#### (Step 7) Selecting behaviour change techniques (BCTs)

The nominated intervention functions and policy categories are then linked with the Behavioural Change Technique taxonomy (BCT Taxonomy version 1) to nominate clear, replicable, and observable intervention content [26]. ‘Forgetfulness’ for instance can be improved with reminders and medication instructions and is represented with the following BCTs: ‘BCT 4.1. Instruction on how to perform a behaviour’ and ‘BCT 8.3. Habit formation’. A total of 18 BCTs (13 for factors related to the person making a quit attempt and 5 for healthcare provider-related factors) were selected based on the nominated intervention functions and policy categories (Table 1).

A comprehensive list of intervention items and functions was discussed among the project team (A.G.M., P.E., M.K., and G.S.G.). The identified intervention strategies were presented to stakeholders using a modified Delphi approach i.e., a two-round email-based survey, described below. Using the identified BCTs, the research team proposed 20 potential intervention strategies (15 targeted to consumers and 5 targeted to health professionals) (Supplementary table 1). Intervention strategies could involve various stakeholders such as the person making a quit attempt, family and friends, health professionals, health care facilities, and policymakers.

#### (Step 8) Selecting mode of intervention delivery

The modes of intervention delivery options were selected using the following two approaches. Considering the lack of evidence on interventions directed to improve adherence to SCMs, an umbrella review was conducted to identify what works for smoking cessation support in general. A systematic review of randomised controlled trials was included to evaluate the effectiveness of various face-to-face and technology-based interventions for improving smoking cessation [33]. The review included five systematic reviews with a total of 212 randomised controlled trials and fourteen intervention modes of delivery including providing a list of websites, peer coaching, social media support using Twitter and Facebook, individually tailored text messages, and interactive phone calls. The interventions were categorised into three broad delivery modes: i) stand-alone web-based, ii) stand-alone mobile phone-based, and iii) multicomponent interventions [33]. Using the umbrella review as a framework, we further evaluated the attitudes of healthcare providers in Australia using a cross-sectional survey. The majority of participants considered face-to-face interventions, mobile phone-based interventions (mobile apps, phone calls, and short text messages), and web-based interventions as acceptable and effective modes to deliver adherence support [34]. Superior effectiveness in smoking cessation was observed when face-to-face approaches were blended with web and/or mobile-based modes of intervention and these modes of intervention were indicated to be acceptable and effective among healthcare providers in Australia to provide SCM adherence support. For instance, the rate of smoking cessation at four weeks and longer was found to be higher when internet-based support was provided in addition to the usual face-to-face behavioural support (RR 1.69, 95% CI 1.30–2.18) [33].

### Stakeholder consultation using modified Delphi study

An online two-round Delphi survey using a purposive sample of experts selected from relevant disciplines was conducted between June and August 2022.

#### Recruitment and sampling

The following types of experts were invited via email: addiction specialists, tobacco researchers, policy members, General Practitioners, and Pharmacists. There are no strict rules on the sample size for Delphi studies [38], however, a minimum of 8 participants is considered adequate to enable consensus [39]. All participants were 18 years or older, English speakers, works in Australia, and have at least 2 years of work experience in their respective professional fields. A total of 31 experts known to the research team were invited to participate in the modified Delphi, 17 experts completed the first round of the survey (response rate of 54.8%) and were further contacted for the second round. In the second-round 14 experts completed the Delphi survey (retention rate of 82.3%). Participants did not receive compensation for participating in the study.

#### Data collection

Open texts were provided to collect information about participant characteristics such as age, profession, professional experience, and type of employment organisation. The participants were presented with proposed intervention strategies. The expert panel has been provided with a review of the existing interventions directed to adherence to SCMs to support their decision-making process and the APEASE criteria with descriptions. During the process, the panellists were asked to indicate their agreement or disagreement with each proposed intervention strategy based on the APEASE criteria, as outlined in the BCW guide, which served as a framework for selecting suitable intervention options [36]. They were also given the option to select 'uncertain' if they were unable to offer an opinion on a particular intervention strategy. Additionally, participants were given the opportunity to provide free-text comments for each intervention strategy, and at the end of the first round, they were invited to suggest any additional intervention strategies they deemed important. Additionally, participants were asked to indicate the minimum intervention strategies that adherence support programmes should include. The top three intervention strategies agreed on by the majority of the experts were selected to be included in the development of an adherence support intervention at a minimum. Data was inputted and analysed using Excel software.

The following definitions of the assessment criteria (the APEASE criteria) were provided along with the intervention strategies [36]:

- *Affordability* – Can the intervention be delivered within an acceptable budget?
- *Practicability* – Can the intervention be delivered as proposed for the target population?
- *Effectiveness* – Can the proposed intervention result in the desired outcome in a real-world context?
- *Acceptability* – Is the intervention deemed appropriate by key relevant stakeholders (public, and professional)?
- *Safety* – Is the intervention safe and has no unwanted side effects or unintended consequences?
- *Equity* – Can the intervention be delivered without increasing the disparities in standard of living, well-being, or health between different sectors of society?

#### Determining consensus

In a Delphi study, a consensus threshold of approximately 70–80% agreement among participants is commonly used [40, 41], although there is no universally defined or fixed percentage that universally indicates consensus. Hence, a consensus threshold of 70% agreement rate was used in this study.

Consensus was defined as follows:

- *Consensus reached and selected as ‘appropriate’*: Intervention strategies where at least 70% of the participants ‘agreed’ for all of the six APEASE criteria. This evaluation method has been successfully employed in previous assessments, using either categorical or scaled evaluations [42, 43].
- *Consensus reached and selected as ‘inappropriate’*: Intervention strategies where less than 50% of experts ‘agreed’ to one or more of the APEASE criteria.
- *Partial consensus reached*: Intervention strategies where 50–70% of the participants ‘agreed’ to one or more of the APEASE criteria.

#### Procedures

Panellists that agreed to participate were emailed the survey and assessment criteria. A two-week deadline was given to complete and return the survey, with a reminder email sent each week if necessary. Consensus was achieved through predefined decision rules to keep, delete, or modify the items.

#### Round one

Intervention strategies where consensus was reached and selected as ‘appropriate’ were included in the final intervention strategy. Intervention strategies where consensus was reached and selected as ‘inappropriate’ were excluded from further consultation. The appropriateness of the intervention strategies for which ‘partial consensus’ had been achieved were amended based on the provided feedback and underwent a second evaluation.

#### Round two

After incorporating the provided feedback, experts were asked to re-evaluate the appropriateness of the strategies for which partial consensus was achieved in the first round. The expert panel's feedback primarily centred around the mode of intervention delivery and the need for clarification on specific intervention strategies. Anonymised group scores from Round 1 were presented for each APEASE criteria of the intervention strategies, and panellists were asked to consider this feedback when rescoring. Appropriateness was assessed as in the first round and intervention strategies meeting agreement by 70% of the panellists on all six APEASE criteria were considered ‘appropriate’ and selected. At the end of Round 2, intervention strategies that still did not meet consensus were excluded.

## Results

### Characteristics of Delphi participants

The Delphi panel (*n* = 17) consisted of two tobacco treatment specialists (*n* = 2); one addiction medicine specialist (*n* = 1); three general practitioners (*n* = 3); two community (*n* = 2) and three hospital pharmacists (*n* = 3); one registered nurse (*n* = 1); two tobacco researchers (*n* = 2); and three behavioural scientists (*n* = 3). The panel incorporated experts working at various academic institutions, Public and private hospitals, general practices, community and hospital pharmacies, National Aboriginal Community Controlled Health Organisations, and health charity organisations. Participants mean age was 47 ± 11 years, ranging from 24 to 63 years. The expert panel had a mean professional work experience of 20 ± 10 years.

### Round one

Among the proposed 20 potential intervention strategies (15 targeted to consumers and 5 targeted to health professionals) (Supplementary table 1), three intervention strategies were selected five intervention strategies were excluded (agreement rate < 50% in any one of the six APEASE criteria). Partial consensus was reached on 12 intervention strategies (agreement rate of 50–70% in one or more of the APEASE criteria). These intervention strategies were amended based on the feedback provided by the experts and formed the second round of evaluation (Table 2).

**Table 2:** Summary of the results obtained in the two evaluation rounds using the APEASE criteria (A-Affordability, P-Practicability, E-Effectiveness and cost-effectiveness, AC-Acceptability, S- safety, EQ-Equity)

Target population	BCT Group	BCTs	Intervention strategies	Appropriateness (Appropriate—A, Inappropriate – I)														Final decision
				**Round 1**							**Round 2**							
				**A**	**P**	**E**	**AC**	**S**	**EQ**	**1**^**st**^** round decision**	**A**	**P**	**E**	**AC**	**S**	**EQ**	**2**^**nd**^** round decision**	
Intervention targeted to consumers offered SCMs	Goals and planning	1.2. Problem solving	Alleviating cues	A	A	A	A	I	I	**Partial consensus**	A	A	A	A	A	A	**A**	**A**
		1.4. Action planning	Action plan	A	A	I	A	I	I	**Partial consensus**	A	A	A	A	A	A	**A**	**A**
	Feedback and monitoring	2.2. Feedback on behaviour	Monitoring and feedback	I	I	I	A	I	I	**Partial consensus**	A	A	A	A	A	A	**A**	**A**
	Social support	3.2. Social support (practical)	Family as a reminder	A	I	I	I	I	I	**I**	-	-	-	-	-	-	**-**	**I**
	Shaping knowledge	4.1. Instruction on how to perform the behaviour	Medication instructions	A	A	A	A	I	A	**Partial consensus**	A	A	A	A	A	A	**A**	**A**
	Natural consequences	5.3. Information about social and environmental consequences	Impact of smoking on Self and family Health	A	A	A	A	A	A	**A**	-	-	-	-	-	-	**-**	**A**
	Repetition and substitution	8.3. Habit formation	Reminders	A	A	A	A	I	I	**Partial consensus**	A	A	A	A	A	A	**A**	**A**
	Comparison of outcomes	9.1. Credible source	Role Model	I	I	I	A	I	I	**I**	-	-	-	-	-	-	**-**	**I**
	Reward and threat	10.1. Material incentive (behaviour)	Access	A	A	A	A	A	A	**A**	-	-	-	-	-	-	**-**	**A**
		10.2. Material reward (behaviour)	Reward	I	I	I	I	I	I	**I**	-	-	-	-	-	-	**-**	**I**
	Regulation	11.1. Pharmacological support	Medication Expectation	A	A	A	A	I	A	**Partial consensus**	A	A	A	A	A	A	**A**	**A**
			Medication Beliefs	A	A	I	I	I	I	**Partial consensus**	A	A	A	A	A	A	**A**	**A**
	Antecedents	12.4. Distraction	Distractions	A	A	I	A	I	I	**I**	-	-	-	-	-	-	**-**	**I**
	Self-belief	15.1. Verbal persuasion about capability	Self-efficacy	A	A	A	A	A	A	**A**	-	-	-	-	-	-	**-**	**A**
			Motivating	I	A	A	I	I	I	**Partial consensus**	A	A	A	I	A	I	**I**	**I**
Intervention targeted to health professionals	Comparison of behaviour	6.1. Demonstration of the behaviour	Training	A	A	A	A	I	I	**Partial consensus**	A	A	A	A	A	A	**A**	**A**
	Reward and threat	10.1. Material incentive (behaviour)	Incentivisation	I	A	I	A	I	I	**Partial consensus**	I	A	I	A	A	A	**I**	**I**
	Shaping knowledge	4.1. Instruction on how to perform the behaviour	Guidelines	A	A	I	A	I	I	**Partial consensus**	A	A	A	A	A	A	**A**	**A**
	Antecedents	12.5. Adding objects to the environment	Quick reference materials	A	A	I	A	I	I	**Partial consensus**	A	A	A	A	A	A	**A**	**A**
	Self-belief	15.1. Verbal persuasion about capability	Persuasion	A	I	I	I	I	I	**I**	-	-	-	-	-	-	**-**	**I**

### Round two

A further 10 intervention strategies were selected and two intervention strategies were excluded (agreement rate < 70% in the second round). The most frequent reasons for exclusion were effectiveness, safety, and equity concerns of the interventions. Finally, from a total of 20 proposed intervention strategies presented to the experts, 13 were selected as appropriate to include in implementation studies and programs targeted at improving adherence to SCMs (Table 2).

### Intervention strategies to improve adherence to smoking cessation medications

In total, thirteen intervention strategies were selected by the expert panel as appropriate to improve adherence to SCMs. Nine BCTs from 8 BCT groups represented by 10 intervention strategies targeted at consumers were selected to be appropriate to improve SCMs adherence. Three BCTs from 3 BCT groups represented by 3 healthcare providers-targeted intervention strategies were selected to be appropriate to improve SCMs adherence.

**Fig. 2:**
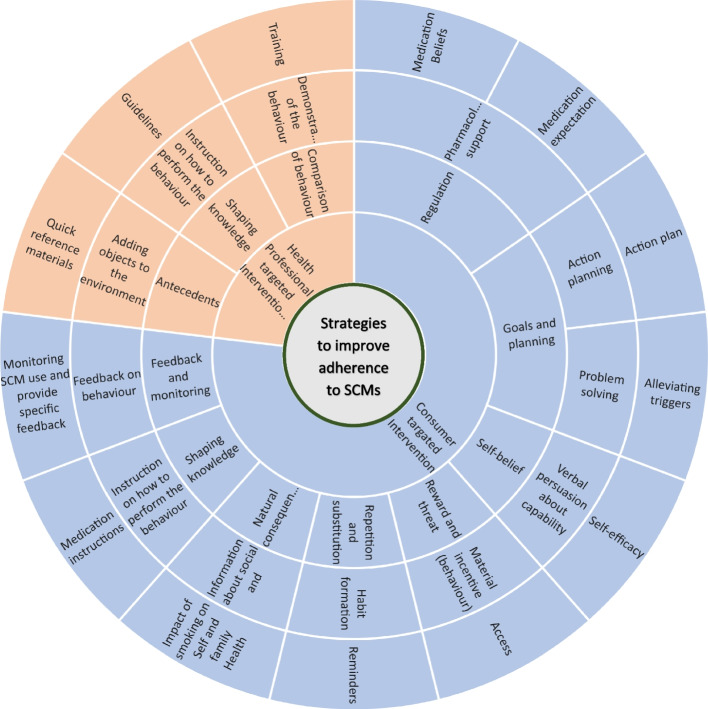
A multilevel adherence support wheel illustrating various strategies selected to improve adherence to smoking cessation medications. Moving from the outer to inner circles of the wheel, the figure shows the selected intervention strategies, behaviour change techniques (BCTs), BCT groups, and the target population. Intervention strategies aimed at healthcare providers are indicated by a light orange shade, while intervention strategies aimed at consumers are represented by a light blue shade

Figure 2 illustrates an adherence support wheel developed based on the findings from this study. Moving from the outer to inner circles of the wheel, we present the 13 selected intervention strategies to be considered in designing and implementing smoking cessation programs targeted at improving adherence to SCMs, BCTs, BCT groups, and the target population. The adherence support wheel incorporated three intervention strategies targeted at healthcare providers, shaded in light orange, and ten intervention strategies targeted at consumers who are offered SCMs to support their quitting, shaded in light blue (Fig. 2).

### Minimum intervention strategies

The majority of participants indicated that for interventions delivered to people making a quit attempt, the following strategies targeted to improve adherence to SCMs should be included at a minimum: providing detailed instruction on how to use the medications; establishing a realistic expectation of the medications; and building self-efficacy that they can quit smoking through encouragement. For interventions targeted to healthcare providers, participants indicated the following interventions to be included at a minimum: prepare guidelines for healthcare providers regarding comprehensive smoking care with specifics on the provision of adherence support tailored for various population groups; quick reference posters and booklets and easy-to-access training.

## Discussion

This study used a combination of evidence in a stepwise manner according to the BCW guide to gain consensus on recommended approaches for interventions to improve adherence to smoking cessation medications. This study provides comprehensive evidence on strategies targeted at improving adherence to SCMs using primary studies and reviews. The study used a pre-defined and comprehensive theoretical framework to indicate potentially effective ways of improving adherence to SCMs. Moreover, a range of factors at multiple levels i.e., consumer-level, healthcare provider level, health facility level, and policy level are included to develop an all-inclusive intervention strategy. This study indicates the need for a comprehensive multi-level approach to improving adherence and enhancing the effectiveness of SCMs.

### Interventions provided to consumers (person making a quit attempt)

#### Education about smoking, smoking cessation, and SCMs

Educating consumers about the health, social, and economic consequences of smoking can be beneficial. Brief intervention strategies that inquire about smoking status, inform individuals about the effectiveness of SCMs, and support quitting journeys can enhance motivation to quit smoking and encourage the appropriate utilisation of SCMs. Detailed explanations of the effectiveness, safety, necessity, and correct ways of using SCMs have been shown to be essential to improve adherence [44]. Providing realistic information regarding the effectiveness of the SCMs or making sure that individuals do not have unrealistic expectations are recommended and can improve adherence. The better way to ensure someone takes their medicine as prescribed is to achieve a partnership-based education on adherence [45]. Education is more effective when it is consumer centred and follows a shared decision-making between consumers and healthcare providers to decide the best way to obtain the desired treatment outcome [46]. Improved information provision and behavioural support focused specifically on medication adherence have shown higher rates of adherence in various clinical conditions such as glaucoma [47] and rheumatoid arthritis [48]. Providing additional information and counselling as an intervention to improve adherence to SCMs has shown inconsistent results [13]. A randomised controlled trial conducted among Chinese adults who smoke in 2011 showed a significant improvement in the rate of quitting at six months in the intervention group who received counselling on medication adherence. However, there was no significant difference in rates of medication adherence [44]. Whereas, brief interventions targeted to establish realistic expectations about the medications and build motivations were found to improve adherence by 2 to threefold as compared to participants receiving standard care [14]. Thus, educational strategies targeted to improve adherence to SCMs are more effective when they are combined with other approaches such as monitoring and feedback [15]. Ruppar et al. suggests that such interventions focused on behaviour change should not rely solely on patient education. Instead, they recommend combining patient education with more active behavioural approaches for optimal results [49].

#### Reminders

One of the main reasons for unintended nonadherence was forgetfulness. This is consistent with previous studies that have also shown that both physical and technological reminders such as alarms and putting medications next to things that the individual uses at least daily (e.g., bathroom mirror or toothbrush) can reduce the risk of forgetting to take medications. Similarly, other studies have shown the importance of supporting individuals to associate SCMs with other daily activities [50]. For example, a randomised trial that included developing a personalised strategy to remember to use nicotine patches (e.g., by linking the patches with brushing teeth) show improved patch use rates in the intervention group [14]. A meta-analysis was conducted in 2012 to evaluate the impact of reminders such as phone calls, text messages, and pagers on medication adherence. The review demonstrated that reminder-based adherence interventions can reduce unintentional non-adherence [50]. Although reminders can improve unintended non-adherence, they should be combined with other adherence intervention strategies to improve reflective motivation and reduce intentional non-adherence [50].

#### Identify and avoid cues for smoking

Encouraging and supporting individuals to identify specific cues for smoking such as being around friends who smoke or drink alcohol in the early stages of a quit attempt, and enabling people to develop clear strategies to alleviate or reduce those cues can improve adherence to SCMs [14]. Various ways of alleviating cues and cravings such as physical activity have been evaluated and shown improved adherence and quit rates [13]. However, this should be combined with other behavioural supports such as referring consumers to multi-session behavioural interventions such as Quitline or health service smoking cessation clinics.

#### Monitoring and feedback

Frequent monitoring of medication use and smoking status is recommended as part of adequate counselling and feedback. Electronic Medication Monitoring cups followed by graphic representations and feedback have shown improvement in medication use and quit rates among clinical trial participants [16, 18]. Furthermore, self-identifying specific strategies that lead to non-adherence such as feelings of stress, anxiety, and depression, and using a collaborative problem-solving strategy to reduce the risk of further non-adherence can improve appropriate medication use [13, 51]. A study conducted in the US indicated significantly higher rates of adherence to bupropion among individuals who were provided with a Medication Event Monitoring System (MEMS) and regular feedback [16]. Similarly, monitoring and feedback improved adherence to nicotine gum in a study conducted in primary care clinics in the US [15]. Combining this strategy with the above-mentioned approaches could provide a more effective way to improve adherence to SCMs.

#### Motivation

Studies have shown the importance of self-efficacy and confidence in the rates of adherence and smoking cessation [14]. A supportive discussion to promote self-efficacy in quitting and making an achievable action plan can improve adherence to SCMs [44]. Motivating individuals through interviewing and presenting relatable role models may improve adherence and quitting rates [9, 51].

#### Medication access

Availing SCMs that are free of cost or subsidised could improve medication adherence. Studies have shown the importance of medication-related costs on adherence, with adherence improvement shown among individuals who accessed medications for free or at a subsidised cost [32].

### Interventions for healthcare providers

#### Guidelines and reference materials

Smoking cessation guidelines are focused mainly on the different treatment regimens for SCMs such as medication dosage [52, 53]. There is a scarcity of evidence-based guidelines on how to provide adequate adherence support for individuals taking SCMs to help their quitting attempts. More comprehensive and detailed guidelines detailing each factor that can lead to non-adherence and how to address them factors are recommended to improve adherence support. In addition to guidelines, quick reference posters or booklets including checkpoints to address during the provision of adherence support that can be placed on healthcare providers' desks may improve adherence support.

#### Training

Evidence-based training on smoking cessation, medication provision, and adherence support is necessary and recommended. Frequent and up-to-date training was found to improve healthcare providers confidence in providing appropriate smoking cessation care [52]. Comprehensive smoking cessation training of healthcare providers was found to improve patient treatment outcomes such as long-term abstinence [53]. Although the effect of healthcare provider training has not been investigated for adherence to SCMs, studies conducted on other medical disorders such as heart diseases and hypertension indicated improved adherence rates and clinical outcomes [54, 55].

Resources to adequately support individuals on their smoking cessation journey such as medications, and quick reference materials can potentially improve the practice of adherence support, medication adherence, and smoking cessation [56, 57]. Therefore, healthcare facilities and policymakers are recommended to work on making the necessary materials easily available and accessible.

Healthcare providers and consumers showed interest in both face-to-face and technology-based interventions. Multi-pronged strategies are recommended for effective smoking cessation, offering flexibility, accessibility, and the chance to build trust. The integration of digital technology, like internet-based cognitive behavioural therapy, with face-to-face therapies is gaining popularity and improving clinical outcomes, including successful smoking cessation [33].

### Strengths and limitations

To our knowledge, this is the first study to use the BCW to develop an intervention directed to improve SCM adherence. A step-by-step systematic approach was used to inform the development of interventions. Six studies were conducted among people who smoke and healthcare providers to provide a more inclusive intervention strategy. One of the limitations of this study is that the designed strategies are general and not contextualised into specific settings or communities but that would not be feasible as the reviews included studies conducted across various communities. While this study offers a comprehensive view of potentially effective intervention strategies, it is important to note that the appropriateness of these interventions is ultimately contingent upon the resources available within specific contexts. Also, the studies were conducted among healthcare providers and people who smoke but did not obtain data from other stakeholders such as health facility managers and policymakers. Furthermore, although people who smoke were not directly engaged in the Delphi process, their perspectives and insights were collected through surveys and carefully considered during the intervention design phase. This approach ensures that their input is incorporated into the development of the interventions.

## Conclusion and recommendations

In this paper, we presented a process for systematically integrating various studies to develop intervention strategies targeted at improving adherence to SCMs. The BCW provided a practical framework to design interventions directed at adherence to SCMs.

Improving adherence to SCMs is a complex issue that requires a comprehensive approach and interventions rather than focusing on a single intervention strategy. Adherence requires a person-centred approach based up on shared decision-making among care providers and consumers. We recommend clinicians to consider this comprehensive list of strategies to ensure they are addressing barriers to SCM use, to improve adherence and cessation rates. It is also recommended that intervention strategies are incorporated into health programs, policies, and clinical guidelines. Further, SCM subsidisation is also recommended to improve medication access, adherence, and smoking cessation. Currently, in Australia, only single-use nicotine replacement therapy is subsidised by the Pharmaceutical Benefits Scheme when evidence suggests that combination nicotine replacement therapy i.e., combining a slower-acting patch with a faster-acting oral form of nicotine replacement therapy is optimal treatment for people who are nicotine dependent.

Although including a wide range of BCTs could result in superior outcomes in enhancing adherence to SCMs, considering the diverse range of resources available within specific contexts it is imperative to tailor and adapt interventions to align with the available resources. This may involve prioritising certain strategies, modifying implementation approaches, or exploring innovative solutions that maximise the impact of interventions while working within resource constraints.

The adherence support wheel could help inform future smoking cessation trials and programs. Future studies are recommended to contextualise the identified intervention strategies for specific communities or population groups and evaluate their acceptability, feasibility, and effectiveness.

## Supplementary Information

**Additional file 1: Supplementary Table 1.** Interventions and intervention descriptions.

## Data Availability

All relevant materials and data supporting the findings of this study are contained within the manuscript. However, if you need additional information, you can access the data from the corresponding author on a responsible request.

## Abbreviations

APEASE: Acceptability, Practicability, Effectiveness, Affordability, Safety/side effect, and Equity
BCW: Behaviour Change Wheel
BCT taxonomy v1: Behaviour change technique taxonomy version 1
BCTs: Behaviour change techniques
COM-B: Capability, Opportunity, Motivation, Behaviour model
SCMs: Smoking cessation medications
