# Supplementary material for: Using the Behaviour Change Wheel and modified Delphi method to identify behavioural change techniques for improving adherence to smoking cessation medications
Source: BMC Public Health. 2023 Jul 17;23:1362. doi: 10.1186/s12889-023-16278-3 (PMC10351182; doi:10.1186/s12889-023-16278-3)
Supplement: Supplementary file 1 — Additional file 1: Supplementary Table 1. Interventions and intervention descriptions. [file 12889_2023_16278_MOESM1_ESM.docx]

**Supplementary Table 1: Interventions and intervention descriptions**

| 1. ***Medication instructions:*** Provide patient with detailed instructions on how to SCMs, frequency of medication use, length of the course of medication, and emphasise the need for the completion of the course. Developing the medication instructions on a community-by-community basis to consider using local languages, consider cultural issues, and prepare in easy-to-understand terms. Make the resources available in various options such as verbal, written, and illustrative materials (such as a leaflet supplied by a pharmacist or other health professionals). Arranging resources to be provided by doctors, nurses, pharmacists, Quitline counsellors. Verbal reinforcement of correct medication use throughout follow-up periods. 2. ***Reminders:*** Helping individuals to link the SCMs with other daily routines such as putting the medications in front of the bathroom mirror, or next to the toothbrush. Supporting individuals to set an electronic reminder that incorporates motivational texts and/or that they can log their smoke-free days. 3. ***Self-efficacy:*** Build self-efficacy that they can quit smoking through encouragement and asking the person to articulate their new identity as an ‘ex-smoker’ can improve their utilisation of SCMs. 4. ***Motivating:*** Motivational interviewing of individuals and arranging for a family member or close friend to motivate the consumers not to go for drinks or other cues that may lead to smoking relapse which affect SCMs use. Motivating appropriate SCM use by weighing the pros and cons of medication through a decisional balance exercise. Build motivation to use the SCMs (weighing the pros and cons of medication use through a decisional balance exercise). Build motivation to use the SCMs (weighing the pros and cons of medication use through a decisional balance exercise). When available, provide training and involve close family members to motivate the consumers not to go for drinks or other cues that may lead to smoking relapse. 5. ***Action plan:*** Support individuals to develop an achievable plan to quit smoking by linking to Quitline or other available resources. 6. ***Distractions:*** Encourage individuals to start other activities such as physical activity to diminish craving and withdrawal symptoms and dismiss the thought of smoking which can affect adherence to SCMs. 7. ***Monitoring and feedback:*** Keep track of and inform the person how far they have come through in the quitting process, and what has been improved since starting the SCMs and quit smoking in a way that empowers individuals and protects patient privacy. Examples: Interactive dashboard or mobile apps for monitoring and feedback on SCM use; daily diary on smoking and medication use followed by tailored feedback and Electronic Medication Monitoring with Feedback giving printouts showing their daily medication use along with counselling sessions focused on the printouts and increasing adherence via collaborative problem-solving. 8. ***Medication expectation:*** Help individuals develop a realistic expectation from the medications and reduce discouragements by helping to understand the extent to which withdrawal symptoms and urges. Help consumers understand the extent to which withdrawal symptoms and urges will be reduced. Individually tailored counselling that considers the type of SCMs and level of nicotine dependence provided by a range of health professionals such as doctors, pharmacists, and Quitline counsellors. 9. ***Medication beliefs:*** Provide education for individuals to improve SCMs safety and efficacy concerns. Comprehensive information about the medications in general and about each product (e.g., short articles about key misconceptions such as overdosing). Detailed information about effectiveness, safety, and necessity of medications. Using printouts, Mobile apps and video resources prepared in plain language to provide this information. 10. ***Alleviating cues:*** Help individuals to identify cues and manage negative emotions such as anxiety, depression, and stress that affect quitting and adherence. Identify personal cues for not using the medications and make plans to avoid or deal with these cues. Examples: reminders, support from family and friends, linking SCMs to the daily routine like brushing teeth; identifying personal cues for not using the medications and developing a plan for avoiding or dealing with these cues; tailoring the medication regime to once usual schedule by associating medication taking with regular activities or routines (e.g., taking other medicines, eating meals, watching a TV show). Linking and motivating individuals to contact Quitline to obtain support to cope with cues and manage negative emotions. 11. ***Role Model:*** Presenting role models who have quit smoking by using appropriate smoking cessation medications to talk about their experience. 12. ***Access:*** Arrange and provide medications for a subsidised cost at health care and policy levels. 13. ***Reward:*** Arrange a financial reward if the medications are used appropriately and the individual stays smoke-free. 14. ***Family as a reminder:*** Inform families and close friends to remind individuals to take the medications appropriately. With patient permission recruit family members/friends and send information sheet home or ask the patient to bring them to the next appointment and discuss how they can help. 15. ***Impact of smoking on Self and family Health:*** Using a holistic approach to educate individuals about the impacts of smoking on the health of the individuals, children living at home and other family members. 16. ***Training:*** Prepare and provide free of cost face-to-face or virtual training prepared in consultation with tobacco treatment specialists and engaging with stakeholders such as Quitline. 17. ***Guidelines:*** Preparing guidelines for health care providers regarding comprehensive smoking care with specifics on the provision of adherence support tailored for various population groups. Engage clinicians in guideline development and make recommendations as clear and actionable as possible with enough details. Engagement can be organised with activities such as workshops to discuss resources such as cost, people, and training requirements. Arrange and provided training of the developed guideline for health care providers. Assess the implementation of the guideline and make necessary amendments. 18. ***Persuasion:*** Persuading HCPs through discussions to create a positive attitude towards adherence support such as perceived role, perceived importance of adherence. 19. ***Incentivisation:*** Integrating incentives for health professionals to provide adequate adherence support such as Medicare Benefits Schedule (MBS) claim item numbers and Continuing Professional Development (CPD) points. 20. ***Quick reference materials:*** Preparing posters and booklets in consultations with clinicians and making the resources available for free. |
| --- |
